# Routinely measured hematological parameters and prediction of recurrent vascular events in patients with clinically manifest vascular disease

**DOI:** 10.1371/journal.pone.0202682

**Published:** 2018-09-07

**Authors:** Daniel Kofink, Steven A. Muller, Riyaz S. Patel, Jannick A. N. Dorresteijn, Gijs F. N. Berkelmans, Mark C. H. de Groot, Wouter W. van Solinge, Saskia Haitjema, Tim Leiner, Frank L. J. Visseren, Imo E. Hoefer, Folkert W. Asselbergs, on behalf of the SMART Study Group

**Affiliations:** 1 Department of Cardiology, University Medical Utrecht, University of Utrecht, Utrecht, the Netherlands; 2 Institute of Cardiovascular Science, Faculty of Population Health Sciences, University College London, London, United Kingdom; 3 The Barts Heart Centre, St Bartholomew's Hospital, London, United Kingdom; 4 Department of Vascular Medicine, University Medical Center Utrecht, University of Utrecht, Utrecht, the Netherlands; 5 Division of Pharmacoepidemiology and Clinical Pharmacology, Utrecht University, Utrecht, the Netherlands; 6 Department of Clinical Chemistry and Hematology, University Medical Center Utrecht, University of Utrecht, Utrecht, the Netherlands; 7 Department of Radiology, University Medical Center Utrecht, University of Utrecht, Utrecht, the Netherlands; 8 Durrer Center for Cardiogenetic Research, ICIN-Netherlands Heart Institute, Utrecht, the Netherlands; Fondazione Toscana Gabriele Monasterio, ITALY

## Abstract

**Background and aims:**

The predictive value of traditional risk factors for vascular events in patients with manifest vascular disease is limited, underscoring the need for novel biomarkers to improve risk stratification. Since hematological parameters are routinely assessed in clinical practice, they are readily available candidates.

**Methods:**

We used data from 3,922 vascular patients, who participated in the Second Manifestations of ARTerial Disease (SMART) study. We first investigated associations between recurrent vascular events and 22 hematological parameters, obtained from the Utrecht Patient Oriented Database (UPOD), and then assessed whether parameters associated with outcome improved risk prediction.

**Results:**

After adjustment for all SMART risk score (SRS) variables, lymphocyte %, neutrophil count, neutrophil % and red cell distribution width (RDW) were significantly associated with vascular events. When individually added to the SRS, lymphocyte % improved prediction of recurrent vascular events with a continuous net reclassification improvement (cNRI) of 17.4% [95% CI: 2.1, 32.1%] and an increase in c-statistic of 0.011 [0.000, 0.022]. The combination of lymphocyte % and neutrophil count resulted in a cNRI of 22.2% [3.2, 33.4%] and improved c-statistic by 0.011 [95% CI: 0.000, 0.022]. Lymphocyte % and RDW yielded a cNRI of 18.7% [3.3, 31.9%] and improved c-statistic by 0.016 [0.004, 0.028]. However, the addition of hematological parameters only modestly increased risk estimates for patients with an event during follow-up.

**Conclusions:**

Several hematological parameters were independently associated with recurrent vascular events. Lymphocyte % alone and in combination with other parameters enhanced discrimination and reclassification. However, the incremental value for patients with a recurrent event was limited.

## Introduction

The most common underlying cause of cardiovascular disease is atherosclerosis, leading to over 13 million deaths per year worldwide [1]. The implementation of preventive therapies critically depends on the reliable identification of individuals at risk. In clinical practice, vascular risk assessment is primarily based on risk factors, such as smoking, hypertension, diabetes, obesity and hyperlipidemia [2]. While a large body of evidence has underpinned the significance of such traditional risk factors in primary prevention [3–5], their predictive value for vascular risk in patients with established vascular disease is less clear [6–8]. Thus, novel risk factors are needed to improve risk stratification in secondary prevention and to establish the pathophysiological processes underlying recurrent vascular risk.

The SMART risk score (SRS) has been specifically developed to predict recurrent vascular events in patients with established atherosclerotic vascular disease [9]. This score not only includes traditional risk factors, but also vascular disease history, renal function and high-sensitive C-reactive protein (hs-CRP), an inflammatory marker associated with vascular risk [10]. Besides hs-CRP, several other biomarkers have been linked to prognosis of vascular disease, including N‐terminal pro‐type brain natriuretic peptide, troponins, ST2 and growth-differentiation factor-15 [6,11]. A recent study identified different routinely-measured hematological parameters that predict outcomes in patients with coronary artery disease [12]. Because these parameters are measured by most hematology analyzers, they are readily available for use in clinical practice without the need to rely on expensive equipment. Despite their potential clinical utility, no study has yet assessed whether hematological parameters improve prediction of recurrent events beyond established secondary risk factors used in the SRS. Combining data from the Second Manifestations of ARTerial Disease (SMART) study and the Utrecht Patient Oriented Database (UPOD), we investigated the incremental value of routinely measured hematological parameters for the prediction of recurrent vascular events. We first investigated associations between 22 hematological parameters and recurrent vascular events. Then, we assessed whether parameters independently associated with recurrent events improved risk prediction compared to the SRS.

## Methods

### Study population

We conducted this study in patients with a clinical manifestation of atherosclerotic vascular disease (cerebrovascular disease, coronary artery disease, peripheral artery disease or abdominal aortic aneurysm) who participated in the SMART study. Details on disease definitions and recruitment procedures have been published previously [9,13]. Briefly, the SMART study, an ongoing, single-center, prospective cohort study, enrolled patients aged 18–80 who were referred to the University Medical Center Utrecht for clinical manifestations of atherosclerotic vascular disease or the treatment of vascular risk factors. Because complete hematological parameters were not available before 2005, we restricted our analysis to a subset of patients enrolled from January 2005 onwards. For this study, follow-up data were available until March, 2014. At baseline, patients were requested to fill in a questionnaire on medical history, symptoms of vascular disease and vascular risk factors. During follow-up, questionnaires were sent to patients or their general practitioner twice a year to obtain information on their health status. Moreover, hospital discharge letters were collected to verify vascular events. All events were adjudicated by three members of the Endpoint Committee. The outcome of interest was a composite endpoint of vascular death, ischemic or hemorrhagic stroke or myocardial infarction, as previously described in more detail [9]. All patients provided written informed consent. The SMART study was approved by the Ethics Committee of the University Medical Center Utrecht.

### Hematological parameters

We enriched the SMART cohort with 22 routinely measured hematological parameters, obtained from UPOD, which comprises clinically relevant data from all patients admitted to the University Medical Center Utrecht, including laboratory measurements. Hematology measurements were performed as part of clinical routine in EDTA blood on the Sapphire hematology analyzers (Abbott, Santa Clara, CA). It uses the multi-angle polarized scatter separation technique. Further details on the quantification of hematological parameters in UPOD have recently been published elsewhere [12].

### Clinical chemistry

Clinical chemistry measurements, i.e. creatinine, total cholesterol, triglycerides, HDL-cholesterol and hs-CRP, were performed in Li-heparin plasma on clinical routine IVD analyzers (AU5800, Beckman Coulter, Brea, CA) at the central diagnostic laboratory of the UMC Utrecht according to international standards (ISO9001, ISO15189). LDL-cholesterol was calculated using the Friedewald equation; eGFR was calculated from creatinine levels according to the MDRD formula.

### Statistical analysis

As for the derivation of the SRS, we truncated all continuous variables, including all hematological parameters, at the 1st and the 99th percentile to reduce the impact of outliers [9]. Using single imputation by additive regression, we imputed missing values for all variables included in the SRS (total n = 126; 0.2%). The variable with the highest percentage of missing values was hs-CRP (n = 75; 1.9%). To facilitate comparison between different hematological parameters, all values were scaled to SD units prior to analysis.

We first evaluated associations between each of the 22 hematological parameters and recurrent vascular events, using Cox proportional hazards modeling adjusted for all SRS variables [age, sex, diabetes mellitus, current smoking, systolic blood pressure, total cholesterol, high-density lipoprotein (HDL) cholesterol, hs-CRP, estimated glomerular filtration rate (eGFR), years since first vascular event, history of cerebrovascular disease, history of coronary artery disease, history of abdominal aortic aneurysm, history of peripheral artery disease]. Analogous to the SRS, hs-CRP was log_e_-transformed and quadratic terms were added for age and eGFR [9]. Since none of hematological parameters showed a skewness >2, log_e_-transformation was not applied. Hematological parameters were entered as quadratic polynomials if the addition of a quadratic term improved model fit, as indicated by the likelihood ratio test (p<0.05). Accordingly, we added a quadratic term for hematocrit. The proportional hazards assumption was tested for each model using scaled Schoenfeld residuals. Associations between hematological parameters and outcome were adjusted for multiple testing. Since several of the 22 parameters were highly correlated (Figure A in S1 File), we estimated the effective number of independent tests for multiple testing correction using principal component analysis. The first 11 principal components explained over 95% of the variance in the hematology data, yielding a significance threshold of 0.05/11 = 0.0045.

We next evaluated the added predictive value of hematological parameters, significantly associated with outcome, by comparing different biomarker models to a reference model in terms of discrimination and reclassification. The reference model was constructed by fitting the SRS variables to our dataset. The single biomarker models included the SRS variables and one of the hematological parameters significantly associated with recurrent event risk. We additionally assessed the performance of multi-biomarker models that included combinations of hematological parameters. To evaluate discrimination, we calculated Harrell’s c for each model and compared c-statistics between each biomarker model and the reference model, using the jackknife approach proposed by Antolini et al [14]. Extending the area under the receiver operating characteristic (ROC) curve to censored outcomes, Harrell’s c measures the ability of a risk prediction model to discriminate individuals with a target events from event-free individuals. Reclassification was assessed by continuous net reclassification improvement (cNRI), as implemented in the nricens R package (https://cran.r-project.org/web/packages/nricens/index.html), which computes NRI for censored survival data. Confidence intervals for NRI were computed by bootstrapping. To obtain robust reclassification indices, we assessed cNRI at 7 years, given a median follow-up of 4.6 years (IQR: 2.5–6.9 years). 7 years also corresponds to the follow-up period for which the SRS was initially calibrated before risk estimates were extrapolated to 10-year risk predictions [9]. Due to the absence of established categories for the 7-year risk of recurrent vascular events, we did not assess categorical NRI.

## Results

3,922 patients with manifest vascular disease enrolled in the SMART cohort were included in this study. Baseline characteristics of the study population are summarized in Table 1. During a median follow-up of 4.6 years (IQR: 2.5–6.9 years), 310 recurrent vascular events occurred. In contrast to Dorresteijn et al. [9], we only included patients recruited from 2005 onwards. Compared to this study, we observed lower event rates (1.7% vs. 2.6%), most likely reflecting improved secondary prevention therapies. In line with this, the proportion of patients treated with statins was higher in our study. Table 2 shows baseline values of all 22 hematological parameters stratified by event status.

**Table 1:** Baseline characteristics.

	All (N = 3922)	No vascular event (N = 3612)	Vascular event (N = 310)
Age, years	61 (54–68)	61 (54–67)	64 (56–71)
Male sex	2850 (73)	2610 (72)	240 (77)
Type of vascular disease			
Cerebrovascular disease	1125 (29)	1032 (29)	93 (30)
Coronary artery disease	2588 (66)	2373 (66)	215 (69)
Peripheral artery disease	531 (14)	481 (13)	50 (16)
Abdominal aortic aneurysm	236 (6)	213 (6)	23 (7)
Years since first vascular event			
less than 1 year	2283 (60)	2140 (61)	143 (48)
1–2 years	389 (10)	363 (10)	26 (9)
over 2 years	1110 (29)	980 (28)	130 (44)
Current smoking	1060 (27)	954 (27)	106 (34)
Diabetes mellitus	704 (18)	628 (17)	76 (25)
Systolic blood pressure, mm Hg	136 (124–149)	135 (124–149)	140 (129–155)
Diastolic blood pressure, mm Hg	80 (73–88)	80 (74–88)	81 (73–90)
eGFR, ml/min/1.73 m^2^	77 (66–88)	77 (67–88)	70 (60–84)
Total cholesterol, mmol/l	4.3 (3.7–5.1)	4.3 (3.7–5.1)	4.3 (3.7–5.1)
LDL cholesterol, mmol/l	2.4 (1.9–3.0)	2.4 (1.9–3.0)	2.4 (1.9–3.1)
HDL cholesterol, mmol/l	1.2 (1.0–1.4)	1.2 (1.0–1.4)	1.1 (1.0–1.4)
Triglycerides, mmol/l	1.2 (0.9–1.8)	1.2 (0.9–1.8)	1.3 (0.9–1.9)
hs-CRP, nmol/l	16 (8–36)	15 (8–34)	26 (12–62)
Medication			
Lipid-lowering drugs	3140 (80)	2888 (80)	252 (81)
Blood pressure-lowering drugs	3086 (79)	2829 (78)	257 (83)
Glucose-lowering drugs	560 (14)	497 (14)	63 (20)
Antithrombotic drugs	3493 (89)	3206 (89)	287 (93)

Discrete variables are expressed as count (%), continuous variables as median (IQR). Type of vascular disease is not mutually exclusive as patients may have experienced several manifestations of vascular disease. eGFR: estimated glomerular filtration rate (see [9]); HDL: high-density lipoprotein; hs-CRP: high-sensitivity C-reactive protein; IQR: inter-quartile range; LDL: low-density lipoprotein.

**Table 2:** Hematological parameters.

	Unit	No vascular event	Vascular event
White blood cells	10^9^/l	6.6 (5.5–7.9)	7.2 (5.9–8.7)
Neutrophils	10^9^/l	3.8 (3.0–4.7)	4.2 (3.5–5.4)
Lymphocytes	10^9^/l	1.9 (1.5–2.4)	1.9 (1.5–2.3)
Monocytes	10^9^/l	0.54 (0.44–0.67)	0.58 (0.49–0.70)
Eosinophils	10^9^/l	0.19 (0.12–0.28)	0.21 (0.15–0.28)
Basophils	10^9^/l	0.04 (0.02–0.06)	0.04 (0.03–0.06)
Neutrophil %	%	57.9 (52.1–63.7)	60.3 (55.1–66.2)
Lymphocyte %	%	29.4 (24.4–34.7)	26.2 (21.8–32.0)
Monocyte %	%	8.2 (6.9–9.7)	8.2 (6.8–9.8)
Eosinophil %	%	2.9 (1.9–4.2)	3.0 (2.1–4.1)
Basophil %	%	0.61 (0.39–0.88)	0.58 (0.36–0.78)
Red blood cells	10^12^/l	4.7 (4.4–5.0)	4.6 (4.2–4.9)
Hemoglobin	mmol/l	8.8 (8.3–9.3)	8.8 (8.2–9.3)
MCV	fl	89.8 (87.1–92.5)	89.9 (86.9–92.8)
RDW	%	12.1 (11.7–12.7)	12.3 (11.8–13.3)
MCH	fmol	1.9 (1.8–2.0)	1.9 (1.8–2.0)
MCHC	mmol/l	21.1 (20.7–21.5)	21.1 (20.5–21.5)
Hematocrit	%	41.7 (39.3–44.1)	41.7 (38.6–44.4)
Platelets	10^9^/l	237 (202–280)	235 (203–276)
MPV	fl	7.7 (7.2–8.4)	7.9 (7.3–8.6)
Plateletcrit	%	0.19 (0.17–0.22)	0.20 (0.17–0.23)
PDW	10xGSD	16.1 (15.8–16.6)	16.2 (15.8–16.6)

Values are expressed as median (IQR) and stratified by event status. GSD: geometric standard deviation; IQR: inter-quartile range; MCH: mean corpuscular hemoglobin; MCHC: mean corpuscular hemoglobin concentration; MCV: mean corpuscular volume; MPV: mean platelet volume; PDW: platelet distribution width; RDW: red cell distribution width.

**Fig 1:**
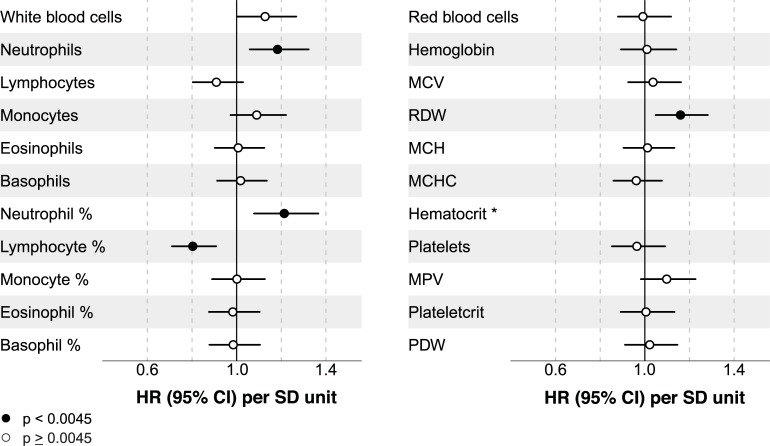
Each of the 22 hematological parameters was analyzed separately. HRs are given per SD-unit increase adjusted for all SRS variables. CI: confidence interval; HR: hazard ratio; MCH: mean corpuscular hemoglobin; MCHC: mean corpuscular hemoglobin concentration; MCV: mean corpuscular volume; MPV: mean platelet volume; PDW: platelet distribution width; RDW: red cell distribution width; SD: standard deviation; SRS: SMART risk score. *A quadratic term was added for hematocrit. Significance test for quadratic polynomial after adjustment for all SRS variables: χ2(df = 2) = 6.2; p = 0.045.

First, we studied associations between hematological parameters and secondary vascular outcomes. Table A in S1 File displays unadjusted and adjusted hazard ratios (HRs) for all hematological parameters. HRs for all SRS variables (reference model) are shown in Table B in S1 File. Since most hematological parameters are directly or indirectly related to immunological processes, we assessed whether these associations were independent of hs-CRP. The addition of hs-CRP particularly attenuated effect estimates for white blood cell count, neutrophil count, monocyte count and neutrophil % (Fig B in S1 File). Four parameters remained significantly associated with vascular events after adjustment for the SRS variables (Fig 1). Lymphocyte % showed a negative association with the recurrent vascular events (HR in SD units: 0.80 [95% CI: 0.71, 0.91]), whereas neutrophil count (HR in SD units: 1.19 [1.06, 1.33]), neutrophil % (HR in SD units: 1.22 [1.08, 1.37]), and RDW (HR in SD units: 1.16 [1.05, 1.28]) were positively associated with recurrent vascular events.

**Fig 2:**
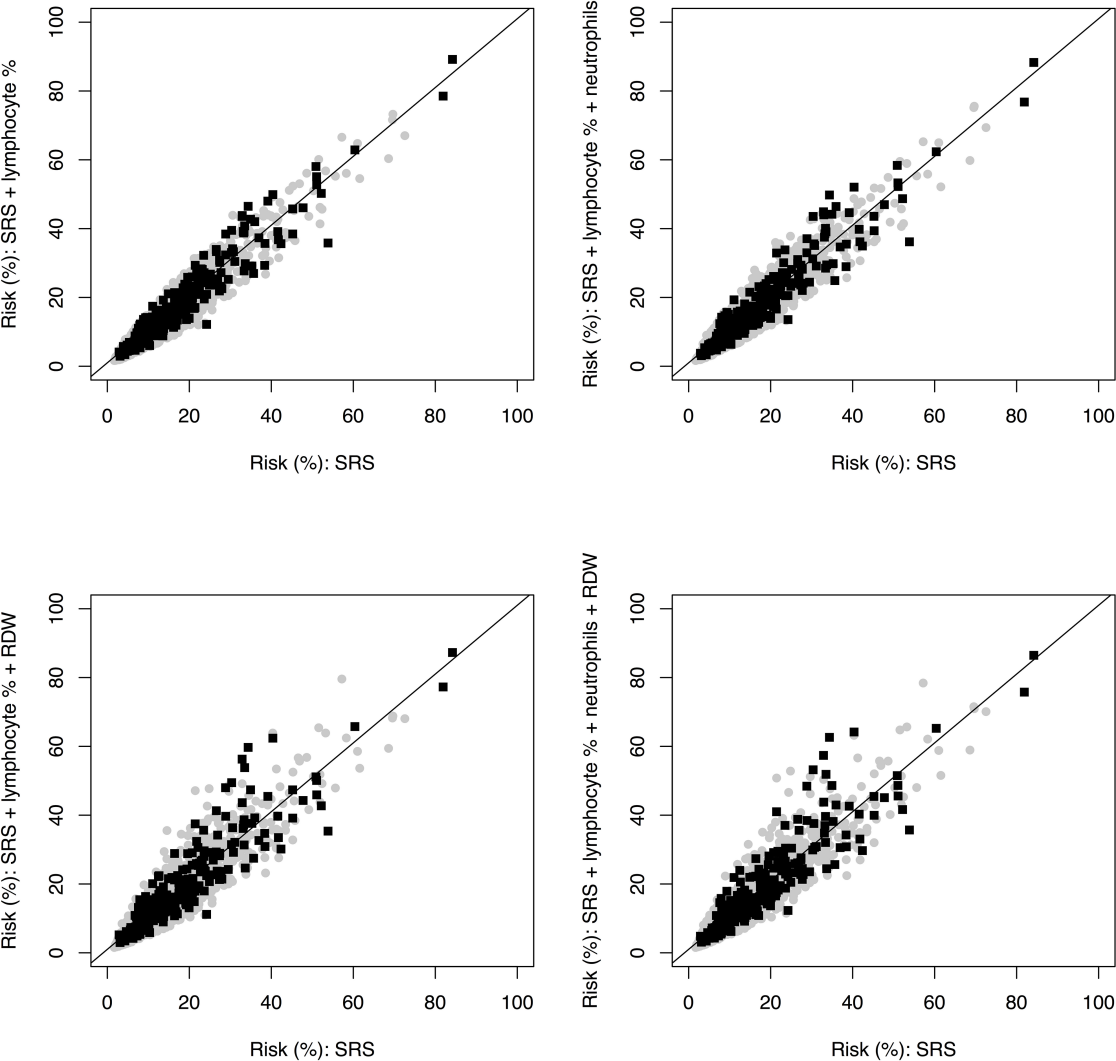
Predicted 7-year risks for reference model (SRS) vs. selected biomarker models (SRS + hematological parameters) stratified by event status. Patients who did not experience a recurrent vascular event during 7-years of follow up (gray circles) were correctly reclassified if there predicted risk was lower after the addition of hematological parameters to the SRS (below the black line). Patients who experienced an event (black squares) were correctly reclassified if there predicted risk was higher after the addition of hematological parameters to the SRS (above the black line). RDW: red cell distribution width; SRS: SMART risk score.

To assess discrimination and continuous reclassification, we next added each of the four hematological parameters that were independently associated with recurrent event risk to a reference model composed of the SRS variables (Table 3). We observed the largest cNRI for lymphocyte %. For events, this parameter improved continuous reclassification by 13.6%, for non-events by 3.8%, yielding a cNRI of 17.4% [95% CI: 2.1, 32.1%]. Additionally, lymphocyte % improved discrimination (c-statistic) by 0.0110 [95% CI: 0.0004, 0.0216]. We also tested whether lymphocyte % combined with other parameters further improved the predictive performance of the SRS. Neutrophil % was not included into a multi-biomarker model because this parameter was highly correlated with lymphocyte % (r = -0.92). Lymphocyte % and neutrophil count improved cNRI by 22.2% [3.2, 33.4%]. The increase in c-statistic was 0.0112 [0.0004, 0.220], which was comparable to that achieved by lymphocyte % alone. For lymphocyte % and RDW combined, the cNRI was 18.7% [3.3, 31.9%], the improvement in c-statistic was 0.016 [0.004, 0.028]. With a cNRI of 17.2% [4.1, 32.8%], all three parameters yielded a lower reclassification improvement than the combination of lymphocyte % and neutrophil count. Lymphocyte % in combination with RDW improved discrimination with an increase in c-statistic of 0.016 [0.004, 0.028]. Fig 2 illustrates the change in predicted risk for different biomarker models, stratified by event status. While lymphocyte % alone and the combination of lymphocyte % and neutrophil count showed the largest continuous reclassification improvement (Table 3) for events, risk estimates increased only modestly in patients who experienced an event. Lymphocyte % and RDW combined predominantly increased risk estimates for events in the higher risk range.

**Table 3:** Predictive performance of hematological parameters.

		Reclassification improvement %		
	Change in c-statistic [95% CI]	with event	without event	Net [95% CI]
Neutrophils	0.006 [-0.002, 0.014]	-9.1	15.6	6.5 [-6.0, 22.7]
Neutrophil %	0.008 [-0.002, 0.018]	7.2	6.7	13.9 [-0.3, 27.7]
Lymphocyte %	0.011 [0.000, 0.022]	13.6	3.8	17.4 [2.1, 32.1]
RDW	0.007 [-0.001, 0.015]	-11.3	25.0	13.6 [-1.9, 26.4]
Lymphocyte % + neutrophils	0.011 [0.000, 0.022]	14.8	7.4	22.2 [3.2, 33.4]
Lymphocyte % + RDW	0.016 [0.004, 0.028]	9.0	9.7	18.7 [3.3, 31.9]
Lymphocyte % + neutrophils + RDW	0.016 [0.004, 0.028]	5.1	12.0	17.2 [4.1, 32.8]

First, hematological parameters significantly associated with outcome were individually added to a reference model composed of the SRS variables. For each single biomarker model (SRS + hematological parameter), we evaluated improvement in discrimination (c-statistic) and reclassification (NRI) compared to the reference model (SRS). We then assessed the predictive performance of multi-biomarker models comprising combinations of lymphocyte % and other hematological parameters. NRI: net reclassification improvement; RDW: red cell distribution width; SRS: SMART risk score.

## Discussion

In this study, we evaluated the incremental predictive value of routinely measured hematological parameters for the prediction of recurrent vascular events in patients with established vascular disease. We first investigated associations between 22 parameters and recurrent event risk and then assessed whether parameters associated with outcome improved risk prediction. Out of the four parameters significantly associated with outcome, lymphocyte % showed the largest cNRI when individually added to the SRS. Overall, the combination of lymphocyte % and neutrophil count yielded the largest cNRI compared to the SRS, but only modestly improved discrimination (c-statistic) and risk estimates for patients who experienced an event during follow-up.

Lymphocytes have been implicated in the modulation of inflammatory processes at distinct stages of atherogenesis [15]. Numerous observational studies in patients with coronary artery disease have reported associations of low absolute and relative lymphocyte levels with poor cardiovascular outcomes [12,16–21]. However, some studies found no link between absolute lymphocyte count and all-cause mortality in pre-existing coronary artery disease [22–24]. Consistent with a role of low lymphocyte levels in vascular disease progression, lymphocyte apoptosis is enhanced in myocardial infarction, but not in stable angina, indicating that low lymphocyte levels may specifically reflect inflammatory processes in advanced atherosclerosis (e.g. plaque rupture) [25]. In our study, however, lymphocyte % rather than absolute lymphocyte count was associated with recurrent vascular events. Accordingly, lymphocyte levels were comparable between patients with and without a recurrent event during follow-up–unlike concentrations of other white blood cell types, such as neutrophils and monocytes (Table 2). Low lymphocyte % may thus reflect increased levels of other white blood cell types in patients at risk.

Besides lymphocyte %, both absolute and relative neutrophil count were independently associated with recurrent vascular risk without improving risk prediction when individually added to the SRS. The combination of lymphocyte % and absolute neutrophil count showed the largest cNRI of all models assessed, but only moderately increased risk estimates for events. The discrimination improvement with lymphocyte % and absolute neutrophil count was likewise limited with an increase in c-statistic equal to that achieved by lymphocyte % alone. The neutrophil to lymphocyte ratio has been widely studied as a marker of cardiovascular risk, suggesting that neutrophil levels are associated with poor prognosis of coronary and peripheral artery disease [26]. There is mounting evidence that neutrophils play an important role in early and advanced atherosclerosis by exacerbating endothelial dysfunction, recruiting monocytes to atherosclerotic lesions, promoting foam cell formation and by destabilizing atherosclerotic plaques [27].

RDW was also independently associated with clinical outcome. Several studies have linked increased RDW to poor outcomes in patients with coronary artery disease, stroke or peripheral artery disease [12,28–31]. RDW is a measure of the variation in erythrocyte volume. The mechanisms by which RDW relates to cardiovascular risk are unknown. Severe inflammation is associated with inhibition of erythrocyte maturation, which results in anisocytosis, suggesting that RDW reflects enhanced inflammation in atherosclerosis, potentially relevant to disease progression [32]. However, RDW did not improve risk prediction and, when combined with lymphocyte %, yielded a cNRI comparable to that achieved by lymphocyte % alone. Moreover, RDW and lymphocyte % predominantly increased risk estimates for events in the higher risk range. Since patients with a high SRS would already be eligible for increased surveillance and more extensive treatment, the added value of RDW for clinical risk prediction is limited.

In the unadjusted analysis, total white blood cell count and monocyte count were strongly associated with recurrent events. However, adjustment for all SRS variables attenuated effect estimates for both parameters, especially due to the inflammatory marker hs-CRP (Fig B in S1 File). In vitro findings suggest that CRP interacts with monocytes to enhance inflammation in acute coronary syndrome [33]. Thus, hs-CRP and monocytes may share a common pathophysiological pathway, whereas other hematological parameters may reflect inflammatory processes that do not, or to a lesser extent, involve CRP. Overall, our findings lend further support to the inflammatory hypothesis of atherothrombosis and add to recent clinical trial data suggesting that anti-inflammatory therapy reduces cardiovascular risk in secondary prevention [34].

Hematological parameters are routinely measured in many hospitals and do not require expensive equipment for analysis, underscoring their clinical potential. In our study, lymphocyte % alone and combined with other hematological parameters yielded the largest cNRI. However, these models only marginally improved discrimination and absolute risk estimates for events. Thus, it remains to be determined whether the incorporation of hematological parameters into risk prediction algorithms would influence clinical decision making in secondary prevention. Since many clinical and demographic characteristics are not assessed systematically in clinical routine, it is often not possible to calculate clinical scores, such as the SRS. Routine hematology testing may be combined with other emerging biomarker technologies suitable for clinical laboratory use to construct biomarker risk scores that do not depend on the availability of clinical information. Such biomarker-based scores could routinely be computed by clinical chemistry laboratories, facilitating the implementation of risk assessment tools for secondary prevention in clinical practice. Besides adding hematological parameters to established clinical scores, future studies also evaluate their predictive value in combination with other biomarkers.

Moreover, the ability of hematological parameters to predict recurrent vascular risk may vary between different manifestations of vascular disease, such as myocardial infarction and ischemic stroke. Since hematological parameters were not available from all SMART patients, the sample size of our study population was limited. As a result, we could not perform stratified analyses for different vascular disease groups. Therefore, further research is required to corroborate our findings in larger cohorts and establish the predictive value of hematological parameters for different manifestations of vascular disease.

In conclusion, we identified several hematological parameters that were independently associated recurrent vascular event in patients with vascular disease. When added to a model comprising the SRS variables, lymphocyte % alone and in combination with other hematological parameters, especially with neutrophil count, improved risk prediction, but only modestly increased risk estimates for patients who experienced a recurrent vascular event.

## Supporting information

S1 File Supporting information. (PDF)

## References

[1] Mendis S, Puska P, Norrving B. Global atlas on vascular disease prevention and control Geneva: World Health Organization, 2011.

[2] Piepoli MF, Hoes AW, Agewall S, Albus C, Brotons C, Catapano AL, et al 2016 European guidelines on vascular disease prevention in clinical practice: the sixth joint task force of the European Society of Cardiology and other societies on vascular disease prevention in clinical practice. Eur Heart J. 2016;37:2315–2381. 10.1093/eurheartj/ehw106 27222591 PMC4986030

[3] D’Agostino RB, Vasan RS, Pencina MJ, Wolf PA, Cobain M, Massaro JM, et al General vascular risk profile for use in primary care. Circulation. 2008;117:743–753. 10.1161/CIRCULATIONAHA.107.699579 18212285

[4] Di Angelantonio E, Sarwar N, Perry P, Kaptoge S, Ray KK, Thompson A, et al Major lipids, apolipoproteins, and risk of vascular disease. JAMA. 2009;302:1993–2000. 10.1001/jama.2009.1619 19903920 PMC3284229

[5] Yusuf S, Hawken S, Ôunpuu S, Dans T, Avezum A, Lanas F, et al Effect of potentially modifiable risk factors associated with myocardial infarction in 52 countries (the INTERHEART study): case-control study. Lancet. 2004;364: 937–952. 10.1016/S0140-6736(04)17018-9 15364185

[6] Beatty AL, Ku IA, Bibbins‐Domingo K, Christenson RH, DeFilippi CR, Ganz P, et al Traditional risk factors versus biomarkers for prediction of secondary events in patients with stable coronary heart disease: from the heart and soul study. J. Am Heart Assoc. 2015;4:e001646 10.1161/JAHA.114.001646 26150476 PMC4608062

[7] D’Agostino RB, Belanger AJ, Kannel WB, Cruickshank JM. Relation of low diastolic blood pressure to coronary heart disease death in presence of myocardial infarction: the Framingham Study. BMJ 1991;303:385–389. 1912805 10.1136/bmj.303.6799.385 PMC1670714

[8] Romero-Corral A, Montori VM, Somers VK, Korinek J, Thomas RJ, Allison TG, et al Association of bodyweight with total mortality and with vascular events in coronary artery disease: a systematic review of cohort studies. Lancet 2006;368:666–678. 10.1016/S0140-6736(06)69251-9 16920472

[9] Dorresteijn JAN, Visseren FLJ, Wassink AMJ, Gondrie MJA, Steyerberg EW, Ridker PM, et al Development and validation of a prediction rule for recurrent vascular events based on a cohort study of patients with arterial disease: the SMART risk score. Heart. 2013;99:866–872. 10.1136/heartjnl-2013-303640 23574971

[10] Buckley DI, Fu R, Freeman M, Rogers K, Helfand M. C-reactive protein as a risk factor for coronary heart disease: a systematic review and meta-analyses for the US Preventive Services Task Force. Ann Intern Med. 2009;151;483–495. 19805771 10.7326/0003-4819-151-7-200910060-00009

[11] Eggers KM, Lindahl B. Prognostic biomarkers in acute coronary syndromes: risk stratification beyond cardiac troponins. Curr Cardiol Rep. 2017;19:29 10.1007/s11886-017-0840-3 28315120 PMC5357245

[12] Gijsberts CM, den Ruijter HM, de Kleijn DP, Huisman A, ten Berg MJ, van Wijk RH, et al Hematological parameters improve prediction of mortality and secondary adverse events in coronary angiography patients: a longitudinal cohort study. Medicine. 2015;94:e1992 10.1097/MD.0000000000001992 26559287 PMC4912281

[13] Simons PC, Algra A, van de Laak MF, Grobbee DE, van der Graaf Y. Second Manifestations of ARTerial disease (SMART) study: rationale and design. Eur J Epidemiol. 1999;15:773–81. 10608355 10.1023/a:1007621514757

[14] Antolini L, Nam BH, D'Agostico RB. Inference on correlated discrimination measures in survival analysis: a nonparametric approach. Commun Statist Theory Meth. 2004;33 2117–2135.

[15] Galkina E, Ley K. Immune and inflammatory mechanisms of atherosclerosis. Ann Rev Immunol. 2009;27:165–197. 19302038 10.1146/annurev.immunol.021908.132620 PMC2734407

[16] Dragu R, Huri S, Zuckerman R, Suleiman M, Mutlak D, Agmon Y, et al Predictive value of white blood cell subtypes for long-term outcome following myocardial infarction. Atherosclerosis. 2008;196: 405–412. 10.1016/j.atherosclerosis.2006.11.022 17173924

[17] Horne BD, Anderson J, John JM, Weaver A, Bair TL, Jensen KR, et al Which white blood cell subtypes predict increased cardiovascular risk?. J Am Coll Cardiol. 2005;45: 1638–1643. 10.1016/j.jacc.2005.02.054 15893180

[18] Núñez J, Sanchis J, Bodí V, Núñez E, Mainar L, Heatta AM, et al Relationship between low lymphocyte count and major cardiac events in patients with acute chest pain, a non-diagnostic electrocardiogram and normal troponin levels. Atherosclerosis. 2009;206:251–257. 10.1016/j.atherosclerosis.2009.01.029 19230894

[19] ó Hartaigh B, Bosch JA, Thomas GN, Lord JM, Pilz S, Loerbroks A, et al Which leukocyte subsets predict cardiovascular mortality? From the LUdwigshafen RIsk and Cardiovascular Health (LURIC) Study. Atherosclerosis. 2012:224;161–169. 10.1016/j.atherosclerosis.2012.04.012 22809446

[20] Ommen SR, Gibbons RJ, Hodge DO, Thomson SP. Usefulness of the lymphocyte concentration as a prognostic marker in coronary artery disease. Am J Cardiol. 1997:79;812–814. 9070569 10.1016/s0002-9149(96)00878-8

[21] Zouridakis EG, Garcia-Moll X, Kaski JC. Usefulness of the blood lymphocyte count in predicting recurrent instability and death in patients with unstable angina pectoris. Am J Cardiol. 2000;86:449–51. 10946041 10.1016/s0002-9149(00)00963-2

[22] Azab B, Shah N, Akerman M, McGinn JT. Value of platelet/lymphocyte ratio as a predictor of all-cause mortality after non-ST-elevation myocardial infarction. J Thromb Thrombolysis. 2012:34(3); 326–334. 10.1007/s11239-012-0718-6 22466812

[23] Azab B, Zaher M, Weiserbs KF, Torbey E, Lacossiere K, Gaddam S, et al Usefulness of neutrophil to lymphocyte ratio in predicting short-and long-term mortality after non–ST-elevation myocardial infarction. Am J Cardiol. 2010;106:470–476. 10.1016/j.amjcard.2010.03.062 20691303

[24] Gijsberts CM, Ellenbroek GH, Ten Berg MJ, Huisman A, van Solinge WW, Asselbergs FW, et al Routinely analyzed leukocyte characteristics improve prediction of mortality after coronary angiography. Eur J Prev Cardiol. 2016;23:1211–1220. 10.1177/2047487315621832 26643521

[25] Pasqui AL, Di Renzo M, Bova G, Bruni F, Puccetti L, Pompella G, et al T cell activation and enhanced apoptosis in non-ST elevation myocardial infarction. Clin Exp Med. 2003; 3:37–44. 10.1007/s102380300014 12748878

[26] Balta S, Celik T, Mikhailidis DP, Ozturk C, Demirkol S, Aparci M, et al The relation between atherosclerosis and the neutrophil–lymphocyte ratio. Clin Appl Thromb Hemost. 2016;22:405–411. 10.1177/1076029615569568 25667237

[27] Soehnlein O. Multiple roles for neutrophils in atherosclerosis. Circ Res. 2012;110;875–888. 10.1161/CIRCRESAHA.111.257535 22427325

[28] Dabbah S, Hammerman H, Markiewicz W, Aronson D. Relation between red cell distribution width and clinical outcomes after acute myocardial infarction. Am J Cardiol. 2010;105: 312–317. 10.1016/j.amjcard.2009.09.027 20102941

[29] Tonelli M, Sacks F, Arnold M, Moye L, Davis B, Pfeffer M. Relation between red blood cell distribution width and cardiovascular event rate in people with coronary disease. Circulation. 2008;117:163–168. 10.1161/CIRCULATIONAHA.107.727545 18172029

[30] Ani C, Ovbiagele B. Elevated red blood cell distribution width predicts mortality in persons with known stroke. J Neurol Sci. 2009;277:103–108. 10.1016/j.jns.2008.10.024 19028393

[31] Ye Z, Smith C, Kullo IJ. Usefulness of red cell distribution width to predict mortality in patients with peripheral artery disease. Am J Cardiol. 2001;107:1241–1245. 10.1016/j.amjcard.2010.12.023 PMC3209662 21296321

[32] Montagnana M, Cervellin G, Meschi T, Lippi G. The role of red blood cell distribution width in cardiovascular and thrombotic disorders. Clin Chem Lab Med. 2012;50: 635–641. 10.1515/cclm.2011.831 22505527

[33] Liuzzo G, Santamaria M, Biasucci LM, Narducci M, Colafrancesco V, Porto A, et al Persistent activation of nuclear factor kappa-B signaling pathway in patients with unstable angina and elevated levels of C-reactive protein: evidence for a direct proinflammatory effect of azide and lipopolysaccharide-free C-reactive protein on human monocytes via nuclear factor kappa-B activation. J Am Coll Cardiol. 2007;49:185–194. 10.1016/j.jacc.2006.07.071 17222729

[34] Ridker PM, Everett BM, Thuren T, MacFadyen JG, Chang WH, Ballantyne C, et al Antiinflammatory therapy with canakinumab for atherosclerotic disease. N Engl J Med. 2017. 10.1056/NEJMoa1707914 28845751

