# Supplementary material for: Routinely measured hematological parameters and prediction of recurrent vascular events in patients with clinically manifest vascular disease
Source: PLoS One. 2018 Sep 7;13(9):e0202682. doi: 10.1371/journal.pone.0202682 (PMC6128486; doi:10.1371/journal.pone.0202682)
Supplement: S1 File — (PDF) [file pone.0202682.s001.pdf]

Supporting Information

Table A: Associations of hematological parameters with recurrent events

Table B: Multivariable associations of SRS variables with recurrent events

Fig A: Correlation heat map of hematological parameters

Fig B: Unadjusted and CRP-adjusted HRs

Table A: Associations of hematological parameters with recurrent events

	Unadjusted		Adjusted for hsCRP		Adjusted for SRS variables	
	HR [95% CI]	p	HR [95% CI]	p	HR [95% CI]	p
White blood cells	1.32 [1.20, 1.45]	<0.0001	1.18 [1.07, 1.32]	0.0018	1.13 [1.00, 1.27]	0.044
Neutrophils	1.38 [1.25, 1.51]	<0.0001	1.25 [1.12, 1.38]	<0.0001	1.18 [1.06, 1.32]	0.0031
Lymphocytes	0.95 [0.85, 1.07]	0.39	0.92 [0.82, 1.03]	0.15	0.91 [0.80, 1.03]	0.13
Monocytes	1.29 [1.17, 1.43]	<0.0001	1.18 [1.06, 1.31]	0.0026	1.09 [0.97, 1.22]	0.14
Eosinophils	1.13 [1.02, 1.25]	0.024	1.08 [0.97, 1.19]	0.17	1.01 [0.90, 1.12]	0.90
Basophils	1.05 [0.95, 1.17]	0.34	1.02 [0.92, 1.14]	0.70	1.02 [0.91, 1.13]	0.75
Neutrophil %	1.39 [1.24, 1.56]	<0.0001	1.28 [1.14, 1.43]	<0.0001	1.21 [1.08, 1.37]	0.0014
Lymphocyte %	0.68 [0.61, 0.77]	<0.0001	0.75 [0.66, 0.84]	<0.0001	0.80 [0.71, 0.91]	0.00048
Monocyte %	1.03 [0.92, 1.15]	0.66	1.04 [0.93, 1.16]	0.50	1.00 [0.89, 1.13]	0.98
Eosinophil %	1.01 [0.90, 1.13]	0.82	1.02 [0.91, 1.14]	0.70	0.98 [0.88, 1.10]	0.77
Basophil %	0.92 [0.83, 1.04]	0.17	0.96 [0.86, 1.08]	0.48	0.98 [0.88, 1.10]	0.79
Red blood cells	0.92 [0.82, 1.03]	0.13	0.98 [0.87, 1.09]	0.68	0.99 [0.88, 1.12]	0.89
Hemoglobin	0.94 [0.84, 1.05]	0.28	1.03 [0.92, 1.15]	0.66	1.01 [0.89, 1.14]	0.88
MCV	1.08 [0.97, 1.21]	0.17	1.09 [0.98, 1.21]	0.13	1.04 [0.93, 1.16]	0.53
RDW	1.30 [1.19, 1.42]	<0.0001	1.21 [1.10, 1.33]	0.00011	1.16 [1.05, 1.28]	0.0039
MCH	1.04 [0.93, 1.16]	0.53	1.06 [0.95, 1.19]	0.26	1.01 [0.90, 1.13]	0.84
MCHC	0.94 [0.84, 1.05]	0.26	0.98 [0.88, 1.10]	0.74	0.96 [0.86, 1.08]	0.49
Hematocrit*						
linear term	1.01 [0.91, 1.12]	0.88	1.07 [0.97, 1.19]	0.19	1.05 [0.94, 1.18]	0.38
quadratic term	1.13 [1.06, 1.22]	0.00032	1.11 [1.00, 1.01]	0.0027	1.09 [1.02, 1.18]	0.014
Platelets	0.99 [0.89, 1.11]	0.89	0.90 [0.80, 1.01]	0.064	0.96 [0.85, 1.09]	0.56
MPV	1.12 [1.01, 1.25]	0.04	1.12 [1.00, 1.24]	0.049	1.10 [0.98, 1.23]	0.10
Plateletcrit	1.03 [0.92, 1.15]	0.57	0.93 [0.83, 1.04]	0.22	1.01 [0.89, 1.13]	0.93
PDW	1.05 [0.94, 1.18]	0.38	1.07 [0.95, 1.19]	0.27	1.02 [0.91, 1.15]	0.71

Table A: Each of the 22 hematological parameters was analyzed separately. HRs for recurrent vascular events are shown unadjusted, adjusted for $\log_e$(hs-CRP) and adjusted for all SRS variables. HRs are given per SD-unit increase. CI: confidence interval; HR: hazard ratio; hs-CRP: high-sensitive C-reactive protein; MCH: mean corpuscular hemoglobin; MCHC: mean corpuscular hemoglobin concentration; MCV: mean corpuscular volume; MPV: mean platelet volume; PDW: platelet distribution width; RDW: red cell distribution width; SD: standard deviation; SRS: SMART risk score.

* A quadratic term was added for hematocrit. Significance test for quadratic polynomial after adjustment for all SRS variables: χ^2(df=2)=6.2; p=0.045.

Table B: Multivariable associations of SRS variables with recurrent events

	HR [95% CI]	df	χ^2	p
Age*	-	2	10.11	0.0064
Sex, male	1.43 [1.07, 1.92]	1	5.81	0.016
History of	-			
cerebrovascular disease	1.40 [1.02, 1.92]	1	4.29	0.038
coronary artery disease	1.16 [0.82, 1.64]	1	0.68	0.41
abdominal aortic aneurysm	1.05 [0.67, 1.64]	1	0.04	0.84
peripheral artery disease	1.06 [0.75, 1.49]	1	0.09	0.76
Time since first vascular event, 10 years	1.22 [1.04, 1.44	1	5.98	0.015
Current smoking	1.70 [1.32, 2.19	1	17.04	<0.0001
Diabetes mellitus	1.26 [0.96, 1.65]	1	2.76	0.097
Systolic blood pressure, 10 mm Hg	1.05 [0.99, 1.11]	1	2.23	0.14
eGFR*	-	2	18.39	0.00010
Total cholesterol, mmol/l	1.06 [0.94, 1.18]	1	0.93	0.33
HDL cholesterol, mmol/l	0.84 [0.58, 1.22]	1	0.84	0.36
$\log_e$[CRP], $\log_e$[nmol/l]	1.35 [1.22, 1.49]	1	32.93	<0.0001

Table B: HRs are given per unit increase. The SRS variables were used to construct a reference model to assess the incremental predictive value of hematological parameters. CI: confidence interval; HR: hazard ratio; eGFR: estimated glomerular filtration rate; HDL: high-density lipoprotein; hs-CRP: high-sensitivity C-reactive protein; LDL: low-density lipoprotein; SD: standard deviation; SRS: SMART risk score.

* A quadratic term was added for age and eGFR. For these variables, df, χ^2 and p refer to the quadratic polynomial.

Fig A: Correlation heat map of hematological parameters

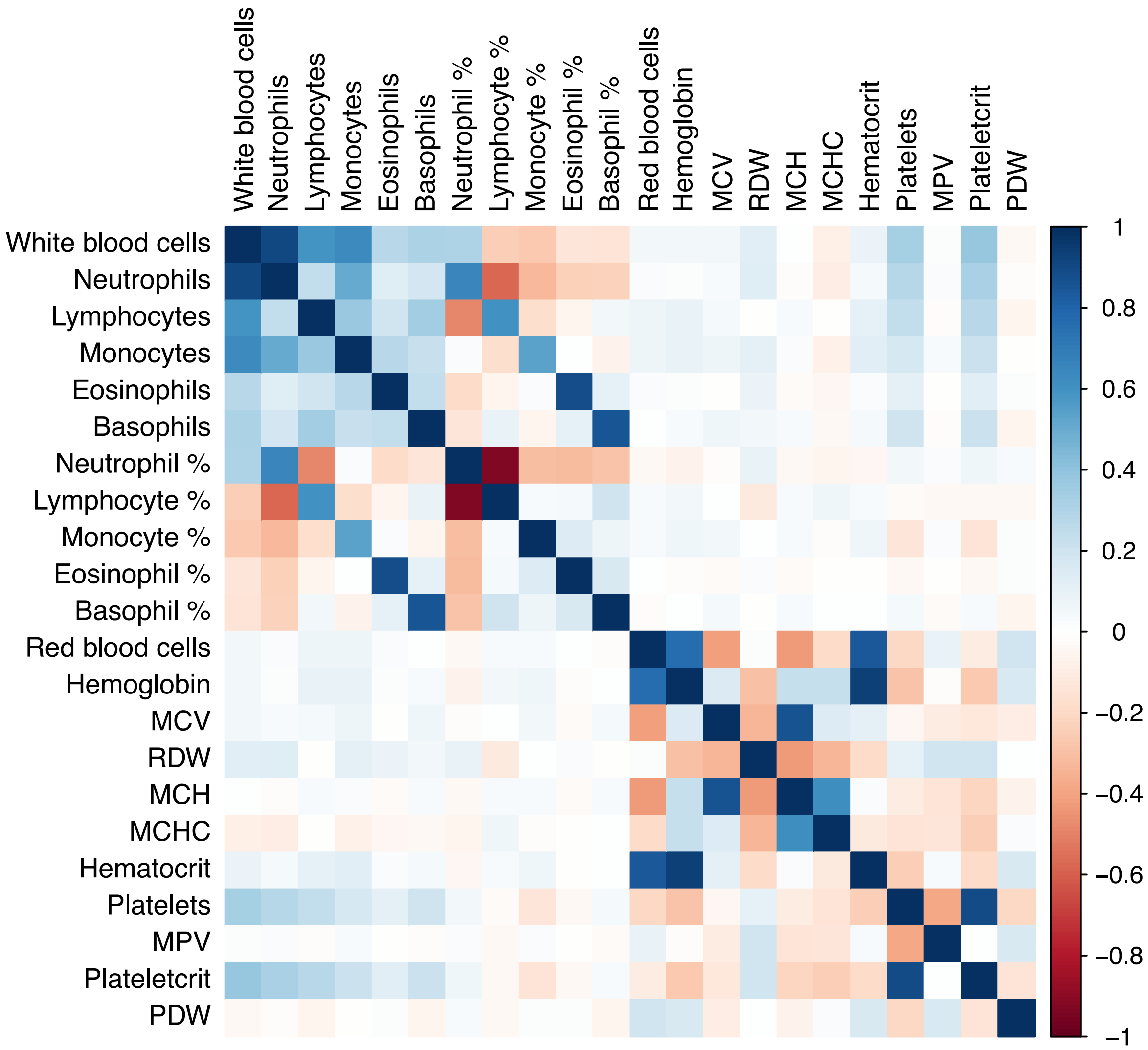

Fig A: Correlation heat map of hematological parameters, Correlations were assessed Pearson's correlation coefficient. MCH: mean corpuscular hemoglobin; MCHC: mean corpuscular hemoglobin concentration; MCV: mean corpuscular volume; MPV: mean platelet volume; PDW: platelet distribution width; RDW: red cell distribution width.

Fig B: Unadjusted and CRP-adjusted HRs

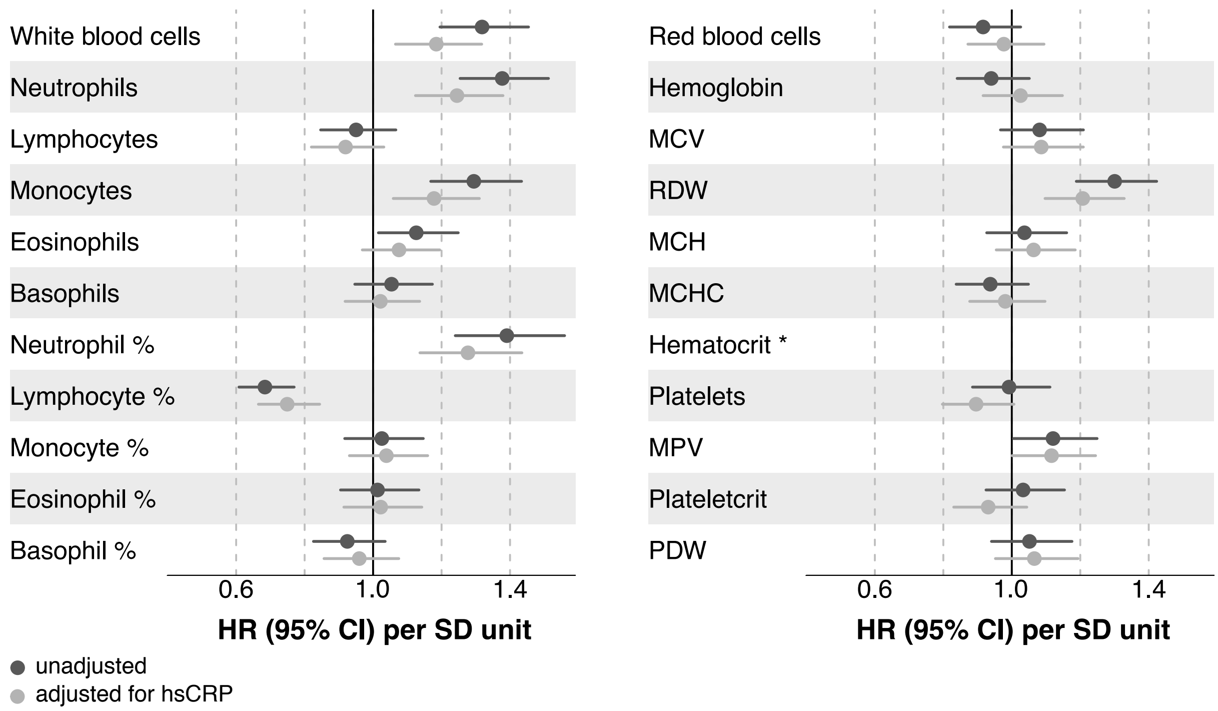

Fig B: HRs for recurrent vascular events are shown unadjusted and adjusted for $\log_e$(hs-CRP). HRs are given in given per SD-unit increase. CI: confidence interval; HR: hazard ratio; MCH: mean corpuscular hemoglobin; MCHC: mean corpuscular hemoglobin concentration; MCV: mean corpuscular volume; MPV: mean platelet volume; PDW: platelet distribution width; RDW: red cell distribution width; SD: standard deviation.

* A quadratic term was added for hematocrit.
